# The Power and Peril of Precise vs. Round Health Message Interventions to Increase Stair Use

**DOI:** 10.3389/fpsyg.2021.624198

**Published:** 2021-07-27

**Authors:** Sebastian Krull, Lea Boecker, David D. Loschelder

**Affiliations:** ^1^Department for Taxation, Accounting and Finance, Center for Risk Management, University of Paderborn, Paderborn, Germany; ^2^Institute of Management and Organization, Leuphana University of Lüneburg, Lüneburg, Germany

**Keywords:** nudging, stair use, health, health risk perception, numeric precision

## Abstract

Taking the stairs vs. an elevator generate benefits for the individual by increasing overall physical activity, health, and wellbeing. In the present paper, we report two pre-registered field intervention studies that examine how health message interventions can motivate individuals to change their behavior. We empirically contrasted opposing predictions from the literature as to whether numerically round (60.00%) or precise (61.87%) health messages are more effective in causing people to use the stairs over taking the elevator. Both interventions were compared to a control condition (no-health message). Contrary to our hypotheses and extant findings, both intervention studies did not produce a significant positive effect of the interventions relative to the control condition. In recent years such null findings have received increasingly more appreciation, particularly in the light of evident downsides of file-drawered studies. We discuss a number of moderating factors that may determine when and why nudging interventions are (in-) effective (e.g., a priori behavioral prevalence, pre-established habits, ceiling effects, and building infrastructure), as well as limitations and avenues for future research.

## Introduction

A more sedentary lifestyle, such as choosing elevators and cars over stairs and bicycles, increases individual’s health risk and impairs wellbeing. The WHO lists physical inactivity as well as a high body mass index (BMI) as death risk factors with an occurrence of 6% and 10%, respectively. High blood pressure is ranked as the number one cause for death with an occurrence of 26% in Germany (World Health Organization, 2005). Sufficient physical activity can decrease the prevalence of hypertension (Diaz and Shimbo, 2013; Hegde and Solomon, 2015) and overweight (Mertens and Van Gaal, 2000). Nudging is a widely respected, promising approach to change behavior by alternating the choice architecture that, in turn, directs people toward more healthier behavior (Sunstein and Thaler, 2008). As there is a plethora of successful nudging interventions across various contexts, nudging is considered a highly suitable soft policy tool to promote healthy lifestyle choices (e.g., Lehner et al., 2016 for an overview of implications).

In the present paper, we build on prior research on choice-architecture interventions and examine a novel research question that contrasts the impact of numerically round (60.00%) vs. precise (61.87%) health messages that foster healthy behavior. Specifically, we report two pre-registered field intervention studies^1^ (Experiment 1 and Experiment 2) that investigate the (in)efficacy of point-of-choice prompts that state health risk reductions by choosing stairs over elevators to alter behavior.

### Physical Activity and Active Transport

For adults, the WHO recommends a minimum of 150 min of moderate-intensity physical activity per week or 75 min of vigorous-intensity activity per week to decrease the risk of non-communicable diseases, such as cardiovascular diseases (World Health Organization, 2010). Based on the BMI, approximately 54% of the German adult population is overweight and about 20% obese (World Health Organization, 2015; Schienkiewitz et al., 2017). In the United States, 2018 even 42% of the adult population was obese (Hales et al., 2020). Although the (dis)advantages of (in)sufficient physical activity are well investigated, surprisingly few actions have been taken. Rare exceptions suggest pragmatic strategies on how to increase the population’s physical activity, such as general improvement of bicycling infrastructure (Jordan et al., 2012) or awareness campaigns (Tuso, 2015).

Stair climbing is a prominent example for physical activity that can be easily integrated into everyday life, with limited time expenses. As most public places and workplaces are equipped with stairs, stair climbing appears to be suitable to increase physical activity without monetary or time expenditure. Increasing physical activity in everyday life seems a suitable approach to countervail current obesity rates. Prior studies have reported success in behavior change toward more physical activity by increasing stair use in a workplace environment (e.g., Kerr et al., 2004; Eves et al., 2006, 2012). In contrast, other studies failed to report an overall positive impact of their intervention (e.g., Coleman and Gonzalez, 2001; Kerr et al., 2001; Avitsland et al., 2017). Thus, the empirical evidence appears inconclusive, and there is little evidence for interventions on stair use for campus environments with a young(er) target population (e.g., Landais et al., 2020). In the present research, we aim at refining evidence on how to shape a successful intervention—to that end, we applied a health message intervention and empirically contrasted the use of numerically precise (vs. round) numbers.

### Risk Communication Using Nudges

From prospect theory (Kahneman and Tversky, 1979; Tversky and Kahneman, 1992), we know that people perceive risks incorrectly. Underestimation of severe health risks potentially undermines sufficient physical activity. Well-designed and credible risk communication is crucial to successfully address risks (Wachinger et al., 2013). But how can credibility of communicated risks be improved? Jenkins et al. (2019) find that communication of numeric estimates for risk is perceived as more credible than verbal probability expressions. Witteman et al. (2011) find that round numeric values are perceived as more reliable than precise numeric values in conveying health risks. However, perceived risk is smaller for round numeric than for precise numeric values, which contradicts the goal of successful risk communication. Another highly relevant factor is the increased sensitivity to relative risk increase compared to absolute risk increase (Furedi, 1999; Gigerenzer et al., 2008; Visschers et al., 2009).

While various choice-architecture interventions have been implemented to increase physical activity by pointing out its benefits on health, few nudging interventions have focused on risk communication to nudge people to increase physical activity. We make use of insights on research on health risk communication to implement these insights into point-of-choice prompts.

### The Efficacy of Numerically Round Vs. Precise Health Messages

There is surprisingly little evidence on the relevance of the numbers used in health risk messages used to nudge people to physical activity. We seek to shed light on the relevance of choosing the most effective numbers to nudge people *via* a health message. Not all numbers are created equal but, instead, are psychologically perceived in different ways—either as precise numbers (e.g., 81.27%) or as round numbers (e.g., 80.00%) with more trailing zeros. Numeric precision coincides with different perceptions of the numbers’ informational content (Loschelder et al., 2013, 2016). Precise information is perceived as an indicator of higher confidence within communication (Welsh et al., 2011), and people are also more likely to follow a precise adviser (Jerez-Fernandez et al., 2014; Schultze and Loschelder, 2020). Recipients expect the communicator to be as accurate and detailed as possible but not more than needed (Grice, 1975; Zhang and Schwarz, 2012).

In contrast, King and Janiszewski (2011) state that people prefer round over precise numbers as responding time for such numbers is lower, indicating a higher processing fluency, which increases liking (Winkielman et al., 2003). Higher precision of numbers (and fewer trailing zeros) inhibits cognitive processing fluency, it increases uncertainty that leads to the favoring of round numbers (Thomas et al., 2010). In a similar vein, Kettle and Häubl (2010) demonstrate that round numbers are likely to increase processing fluency and velocity due to greater frequency in language.

In sum, competing predictions emerge from the literature: Rounded numbers might “feel right” when a decision is based on emotions (Wadhwa and Zhang, 2015). If one considers the choice for or against stair use as an emotional act or habitual behavior, rounded numbers should be more effective for health message interventions than precise numbers. In contrast, if numeric precision effectively evokes the perception of increased competence and accuracy (e.g., Schultze and Loschelder, 2020), health risk message interventions with precise numbers (e.g., “stairclimbing decreases heart problems by 61.87%”) could be more effective than round-numbered interventions (e.g., “heart problems decrease by 60.00%”).

## Study Goals and Hypotheses

We aim at replicating and shedding light on (partially) inconclusive results from studies that applied nudges to increase stair use by using the setting of a German University Campus (e.g., Kerr et al., 2004; Eves et al., 2006, 2012; Müller-Riemenschneider et al., 2010; Rogers et al., 2010; Burger and Shelton, 2011; Lewis and Eves, 2012; Graham et al., 2013). Replications in the field of nudging are highly important to reveal the true potency of nudges given that replication attempts often fail to replicate previous seminal findings (see Scheibehenne et al., 2016; for a meta-analysis). DellaVigna and Linos (2020) show that nudging interventions published in academic journals in comparison with interventions by so-called nudge units (i.e., private or publicly funded organizations that implement behavior change interventions based on the nudging approach) differ markedly in effect size. Scientific studies report an average impact of nudges of 8.7%, while nudge units report real-world effects of only 1.4%. This difference in effect size may be largely explained by publication bias that favors large and significant effects over studies with only a small or even null effect (DellaVigna and Linos, 2020).

A further aim of the present work is to investigate for the first time whether round or precise numbers differ in their effectiveness when integrated into choice-architecture interventions. We term this combination *round* vs. *precise health risk message*, respectively. Prior theorizing allows for competing predictions for round or precise health message. The results offer insights for the design of more effective nudges to foster healthy behavior.

To sum up, we hypothesize (1) an increase in stair use during the nudge intervention phase for both health message conditions compared to baseline, whereas there should be no change in the control condition. With respect to competing predictions for numeric precision, (2a) a higher increase in stair use should emerge for the round than for the precise health risk message condition. Alternatively, (2b) a higher increase in stair use could also emerge for the precise (vs. round) health risk message condition, if precision indeed conveys informational accuracy and credibility.

## Experiment 1

We first investigated whether a nudge in form of a poster placed at the point-of-choice displaying a health risk message would decrease the use of elevators compared to (1) a baseline phase and (2) a no-intervention control group. The health message communicated a reduction in health risk, which has been shown to have a beneficial impact on physical activity (Janssen et al., 2018). In addition, we empirically contrasted round vs. precise messages. We included a follow-up phase (without posters) to investigate the durability of effects. The effect of health messages on elevator use should result in an interaction effect of phase and intervention type.

### Methods

#### Design

Experiment 1 realized a 3 (Phase: baseline vs. intervention vs. follow-up) × 3 (Intervention: precise vs. round health risk message vs. control group) design with daily elevator rides as the dependent variable.

#### Participants and Study Setting

We chose three campus buildings of the Leuphana University of Lüneburg that are largely identical in their architecture. In order to omit a spill-over effect, the buildings were chosen from different faculties. As we conducted an observational study, we did not approach participants to assess demographic variables. The experimental conditions were randomly assigned to the university buildings. The stairwells are well visible upon entering all three buildings, with the elevators slightly around a corner (see further details in the Supplementary Material).

We conducted a post-hoc power analysis in G*Power 3.1 (Faul et al., 2007) for a repeated measures ANOVA using these parameters: three measurements (baseline vs. intervention vs. follow-up), three conditions (precise health risk message vs. round health risk message vs. control condition), *α* = 0.05, a moderate assumed population effect size of *f* = 0.25 (Cohen, 1992), and an assumed conservative correlation between baseline and intervention measurement of *r* = 0.2. Accordingly, the present study was powered at 1−*β* = 98.87%, with a total of 23,766 elevator rides.

#### Material and Procedure

We placed two signs with round (precise) health risk messages on each floor in the intervention buildings at the elevator doors and walls between elevators and stairs, i.e., the point-of-choice. The signs contained the health risk message: “Only 7 min of stairclimbing helps to reduce your risk for a heart attack by 60% (round) vs. 61.87% (precise)” (see Supplementary Figures 3, 4). We based our message on the finding that men whose daily level of vigorous intensity leisure activity equals an average of 7 min stair climbing have a 62% reduction in coronary death (Yu et al., 2003).

We collected data for 36 days—16 days baseline phase, 10 days intervention, and 10 days follow-up (no intervention) for all experimental conditions. The study took place during lecture time of the academic year to ensure consistent visitor traffic. We collected data for objective elevator use by daily reading out the meters integrated into elevators. Reading out took place daily around 9.00 am to ensure a constant measurement interval. In total 23,766 elevator rides were measured, which were all included into subsequent analyses.

### Results

**Figure 1:**
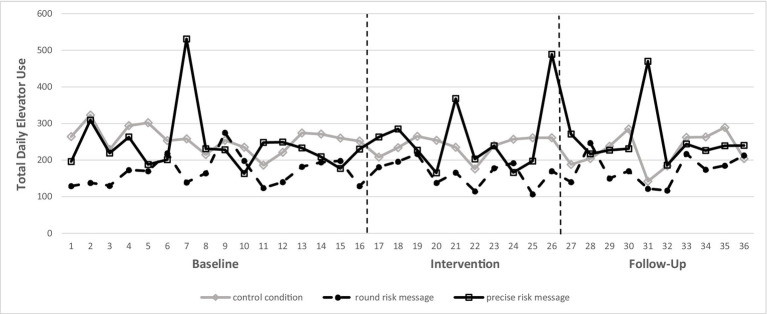
Illustration of elevator use over baseline period, intervention period, and follow-up period.

We conducted a 3 (Phase: baseline vs. intervention vs. follow-up) × 3 (Intervention: precise risk message vs. round risk message vs. control group) ANOVA with repeated measures for the first factor. The 3 × 3 ANOVA revealed a highly significant main effect for buildings, *F* (2, 99) = 19.175, *p* < 0.001, showing that there were overall differences in absolute elevator use between the different intervention buildings. Contrary to our hypotheses, however, there was no significant effect for phase, *F* (2, 99) = 0.027, *p* = 0.974, and no significant interaction of Phase × Intervention, *F* (4, 99) = 0.393, *p* = 0.813. Thus, there were no differences in elevator rides over the different phases of the experiment and neither of our interventions differed relative to the baseline phase and the control group (see Figure 1).

### Discussion

The results for Experiment 1 did not reveal a positive effect for either of our interventions. Instead, we observed highly consistent and robust elevator traffic. As the daily total of elevator rides served as the dependent variable for our analysis, we cannot account for who (and how many individuals) used the elevator, nor for how many participants elected to use the stairs instead. Data on stair use are missing for this experiment; hence, it may be premature to conclude that there was no increase in total building traffic. To address this shortcoming, we decided for a second intervention experiment at the same campus that quantified both absolute elevator rides and stair use to allow for detecting potential changes in the stairs-vs.-elevator-use ratio.

## Experiment 2

Experiment 2 tested whether health message interventions would increase stair use rate relative to (1) a baseline period and (2) a control group—again, we contrasted numerically round vs. precise health risk message interventions.

### Methods

#### Design

Experiment 2 realized a 2 (Phase: baseline vs. intervention) × 3 (Intervention: precise health risk message vs. round health risk message vs. control group) design with relative stair-vs.-elevator use as the key dependent measure.

#### Participants and Study Setting

We kept the study setting as close as possible to Experiment 1. However, the dependent variable addressed limitations of Experiment 1. We chose three different campus buildings to target different participants than in Experiment 1. Again, we randomly assigned experimental conditions to buildings. A post-hoc power analysis in G*Power (Faul et al., 2007) for a repeated measures ANOVA with two measurements (baseline vs. intervention), three intervention conditions (precise vs. round health messages vs. control condition), *α* = 0.05, a moderate effect size of *f* = 0.25 (Cohen, 1992), and an assumed conservative correlation between measurements of *r* = 0.2 showed that the study was powered at 1−*β* = 76.53%.

#### Material and Procedure

To allow comparability, we used the same posters and health messages as in Experiment 1 (see Supplementary Figures 3, 4). We collected data for 20 days – 10 days baseline and 10 days intervention for all experimental conditions. All measurements took place during the semester break, which allowed us to count and to closely observe individuals’ decisions more accurately (due to overall lower traffic). Measurement took place by in-situ observations on the ground floor for 1 h per building daily at randomized times between 8.00 am and 12.00 am. The number of individuals taking the elevator vs. stairs was counted. All observations took place by a carefully instructed observer to avoid potential differences in counting behavior of different observers. We assume no experimenter demand effects, as (1) having persons sitting in the hallway is not unusual at the chosen campus and (2) the trained observer stated that he was barely noticed by most people. In total, 1,497 stair walks and 177 elevator rides were observed. Due to our pre-defined exclusion criteria, individuals with visible disabilities or obviously heavy luggage, 30 elevator rides were excluded. Therefore, 1,497 stair uses and 147 elevator rides were subjected to final analyses. Retaining these data in our analyses did not change the pattern of results.

### Results

We conducted a 2 (Phase: baseline vs. intervention) × 3 (Intervention: precise vs. round health message vs. control group) ANOVA with repeated measures for the first factor. The 2 × 3 ANOVA revealed no significant main effect for Phase, *F* (1, 54) = 0.752, *p* = 0.390, showing that intervention phase did not differ from the baseline period. Contrary to our hypotheses, there was no effect of intervention, *F* (2, 54) = 0.075, *p* = 0.928, and no interaction of Phase × Intervention, *F* (2, 54) = 0.523, *p* = 0.595. Thus, the (precise and round) health risk message interventions did not alter the ratio of stairs-vs.-elevator use relative to baseline period and the no health message control condition.

### Discussion

**Figure 2:**
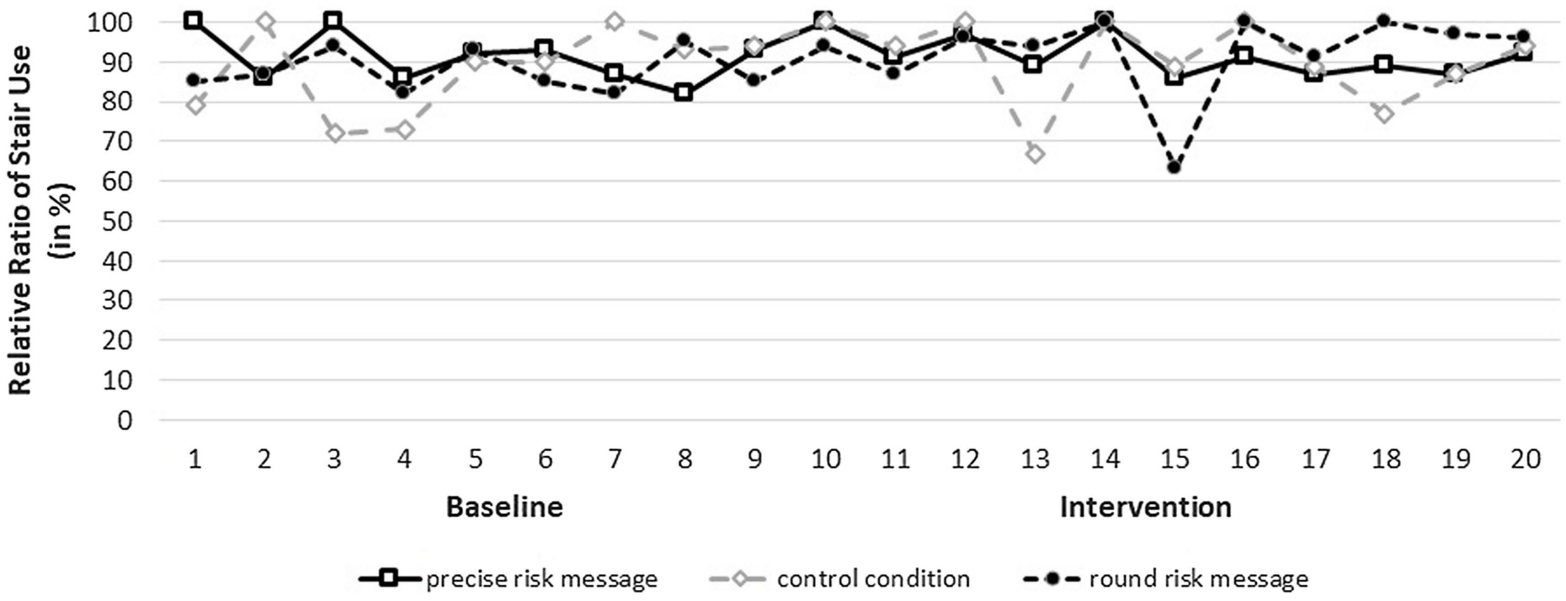
Illustration of stair use rates over baseline period and intervention period.

The results of Experiment 2 did not reveal the predicted, positive effect(s) of our nudging interventions. In contrast, stair use was constant and surprisingly high across all study sites for the entire duration of the experiment, i.e., baseline and intervention phase (see Table 1 and Figure 2). By observing elevator vs. stair use, we controlled for the extent of traffic in the study sites during our experiment. Although we thereby addressed the evident limitations of the dependent measure in Experiment 1, we did not find the predicted significant effects for our health risk message interventions in Experiment 2 either.

**Table 1:** Mean stair use rates for intervention conditions.

	Baseline		Intervention	
	*M* %	*SD* %	*M* %	*SD* %
No intervention control condition	89.1	10.7	89.8	10.8
Precise health risk message condition	91.8	6.6	90.8	4.2
Round health risk message condition	88.23	5.1	92.3	11.3

## General Discussion

The present work aimed at replicating the effect of nudging people to increase stair use to facilitate a higher level of physical activity in everyday life. Expanding prior research, we examined for the first time a potential difference between numerically round and precise health risk messages in a nudging framework. We expected a significant decrease in elevator use (and increase in stair use) for the two health message conditions relative to (1) the baseline phase and (2) the no-intervention control condition. We (3) also contrasted competing predictions regarding whether round or precise health message would exert a stronger effect on physical activity.

Contrary to our hypotheses, our experimental manipulations did not produce a significant effect on stair (vs. elevator) use relative to the baseline period and control condition. There are several possible explanations for our null results: (1) individuals may have had pre-established habits that are difficult to alter with nudging interventions (see De Wijk et al., 2016), (2) unknowingly, the behavior that we aimed to change already constituted the (highly prevalent) default option in this study environment (i.e., a ceiling effect), (3) there is likely a high consciousness for healthy behavior in the studied population, (4) the infrastructure of the buildings in our study may have already favored (and nudged) the use of stairs, and (5) the sign displaying the nudge may have been ineffective because it was either not appealing or not sufficiently attended to.

First of all, we like to highlight that we do not consider it likely that the poster intervention itself was ineffective because the displayed signs were not appealing or did not catch the attention of our participants. Eves et al. (2006) showed that a simple poster intervention can successfully increase stair use. We considered findings on sensitivity to perception of relative health risks (Visschers et al., 2009) and to risk reduction (Janssen et al., 2018). Finally, we used specific health risk messages including numbers (round and precise), which have been shown to be more effective than unspecific messages (Puig-Ribera and Eves, 2010). We believe that other factors that concern the investigated population and the infrastructure are more plausible explanations for our null effects.

Nudging interventions *via* health risk messages aim at changing current behavior and habits toward a societally desired behavior, i.e., improved individual health. However, if the targeted behavior already constitutes the norm, it becomes very difficult to observe additional, incremental benefits due to the presence of a so-called “ceiling effect.” The data of Experiment 2 in particular indicate that the to-be-changed behavior was already the norm as more than 90% percent of all participants already used the stairs (and decided to not use the elevator). Supporting this ceiling effect argument, the measured level of stair use was much higher than in other studies (e.g., Eves et al., 2006). This may be due to the fact that the university where we conducted the present studies puts a distinct emphasis on sustainability-related subjects that overlap in individual and public health. It appears plausible that students and employees who chose the university are aware of health- and sustainability-related topics and may therefore use stairs instead of elevators.

Apart from these person-related factors, the architectural infrastructure of the sites that we used may also account for the high percentage of stair users. The buildings may, in fact, be constructed in a way that (inadvertently) nudges stair use instead of elevator use. An infrastructural nudging influence may have been at play in our studies as individuals enter the buildings right into the stairwell, while the elevators are not highly visible at first glance (see Supplementary Figures 1, 2, 5). Centrality and accessibility of stairs constitute key factors for individuals to use them (e.g., Nicoll, 2007; Bassett et al., 2013).

As outlined in the introduction, the literature offers different and competing predictions regarding whether precise or round numbers more effectively foster stair (vs. elevator) use. For now, the present pattern of results leads to the conclusion that the numerical precision of a health risk message is not substantial. This empirical null finding should be treated with caution, however, as (1) the evident ceiling effect in Experiment 2 may have masked any potential difference in round vs. precise risk messages and/or (2) the true difference between precise and round message interventions may be smaller than the moderately sized effect for which we powered, rendering future research necessary to illuminate these competing predictions (and underlying mechanisms).

Assessing the effect of our health risk messages on the individual level would allow for a nested multilevel model, which we cannot use due to our coarse data measurement. We refrained from identifying individuals for ethical reasons and did not reach out to volunteering individuals to reveal the true effect of our intervention on the sample level and to omit a self-selection bias. While we cannot fully rule out that the observed (null-) effect is driven by specific individuals, we consider this highly unlikely: A spill-over effect between our experimental conditions is unlikely as buildings from different faculties were chosen. In any case, this is of relatively minor interest as we observe a ceiling effect, which is the strongest explanation for our (null-) findings. Nonetheless, future research may further assess the (in-) efficacy of nudging interventions on an individual level to control for further unpredicted, interpersonal factors (see, e.g., Vetter and Kutzner, 2016; Venema et al., 2019; Raghoebar et al., 2020).

As a first step, future studies could present participants with both types of health risk messages and assess participants’ willingness to use stairs as a function of these health risks. Follow-up studies could illuminate the underlying processes accounting for a differential effectiveness of round or precise health risk message. Apart from this, future studies should attempt to study the effect of health risk messages in settings, in which both options (e.g., stairs vs. elevator) are similarly visible and attractive—without an architecturally built-in stair nudge. To assure that the targeted behavior does not yet constitute the prevalent norm, researchers should certainly approximate the percentage of the desired behavior in a brief pilot study. In conclusion, future studies are needed to conclusively disentangle whether the present findings (1) constitute a true null effect that—for problematic file-drawer distortions (Friese and Frankenbach, 2019)—should not disappear in our file drawer or (2) solely emerged because of unforeseen ceiling effects, for which our risk message interventions did not succeed to facilitate incremental benefits in terms of elevated health behavior.

## Data Availability Statement

The datasets presented in this study can be found in online repositories. The names of the repository/repositories and accession number(s) can be found in the following link: https://osf.io/t3b96/?view_only=0064dbb0a69346c898328bf12c99f160.

## Ethics Statement

Ethical review and approval were not required for the study on human participants in accordance with the local legislation and institutional requirements. Written informed consent for participation was not required for this study in accordance with the national legislation and the institutional requirements.

## Author Contributions

All authors contributed equally and qualify for authorship of the paper. All authors read and approved the final manuscript.

## Conflict of Interest

The authors declare that the research was conducted in the absence of any commercial or financial relationships that could be construed as a potential conflict of interest.

## Publisher’s Note

All claims expressed in this article are solely those of the authors and do not necessarily represent those of their affiliated organizations, or those of the publisher, the editors and the reviewers. Any product that may be evaluated in this article, or claim that may be made by its manufacturer, is not guaranteed or endorsed by the publisher.

## Footnotes

^1^Diverging from our pre-registrations we did not examine the effect of (descriptive) social norms as we originally stated. We thank our reviewers for reminding us that we have diverged from our original goal during our research process.
